# Invasiveness of previous treatment for peripheral arterial disease and risk of adverse cardiac events after coronary stenting

**DOI:** 10.1007/s12928-024-00986-7

**Published:** 2024-02-14

**Authors:** Tineke H. Pinxterhuis, Clemens von Birgelen, Robert H. Geelkerken, Carine J. M. Doggen, Theo P. Menting, K. Gert van Houwelingen, Gerard C. M. Linssen, Eline H. Ploumen

**Affiliations:** 1 Department of Cardiology, Thoraxcentrum Twente (A25), Medisch Spectrum Twente, Koningsplein 1, 7512 KZ Enschede, The Netherlands; 2 Department of Health Technology and Services Research, Faculty BMS, Technical Medical Centre, University of Twente, Enschede, The Netherlands; 3 Department of Vascular Surgery, Medisch Spectrum Twente, Enschede, The Netherlands; 4 Department of Multi-Modality Medical Imaging (M3I) Group, Faculty of Science and Technology, Technical Medical Centre, University of Twente, Enschede, The Netherlands; 5 Department of Cardiology, Ziekenhuisgroep Twente, Almelo and Hengelo, The Netherlands

**Keywords:** Coronary artery disease, Percutaneous coronary intervention, Drug-eluting stent, Peripheral arterial disease

## Abstract

Patients with peripheral arterial disease (PADs), undergoing percutaneous coronary intervention (PCI), have higher adverse event risks. The effect of invasiveness of PADs treatment on PCI outcome is unknown. This study assessed the impact of the invasiveness of previous PADs treatment (invasive or non-invasive) on event risks after PCI with contemporary drug-eluting stents. This post-hoc analysis pooled 3-year patient-level data of PCI all-comer patients living in the eastern Netherlands, previously treated for PADs. PADs included symptomatic atherosclerotic lesion in the lower or upper extremities; carotid or vertebral arteries; mesenteric arteries or aorta. Invasive PADs treatment comprised endarterectomy, bypass surgery, percutaneous transluminal angioplasty, stenting or amputation; non-invasive treatment consisted of medication and participation in exercise programs. Primary endpoint was (coronary) target vessel failure: composite of cardiac mortality, target vessel-related myocardial infarction, or clinically indicated target vessel revascularization. Of 461 PCI patients with PADs, information on PADs treatment was available in 357 (77.4%) patients; 249 (69.7%) were treated invasively and 108 (30.3%) non-invasively. Baseline and PCI procedural characteristics showed no between-group difference. Invasiveness of PADs treatment was not associated with adverse event risks, including target vessel failure (20.5% vs. 16.0%; HR: 1.30, 95%-CI 0.75–2.26, *p* = 0.35), major adverse cardiac events (23.3% vs. 20.4%; HR: 1.16, 95%-CI 0.71–1.90, *p* = 0.55), and all-cause mortality (12.1% vs. 8.3%; HR: 1.48, 95%-CI 0.70–3.13, *p* = 0.30). In PADs patients participating in PCI trials, we found no significant relation between the invasiveness of previous PADs treatment and 3-year outcome after PCI. Consequently, high-risk PCI patients can be identified by consulting medical records, searching for PADs, irrespective of the invasiveness of PADs treatment.

**Graphical abstract:**

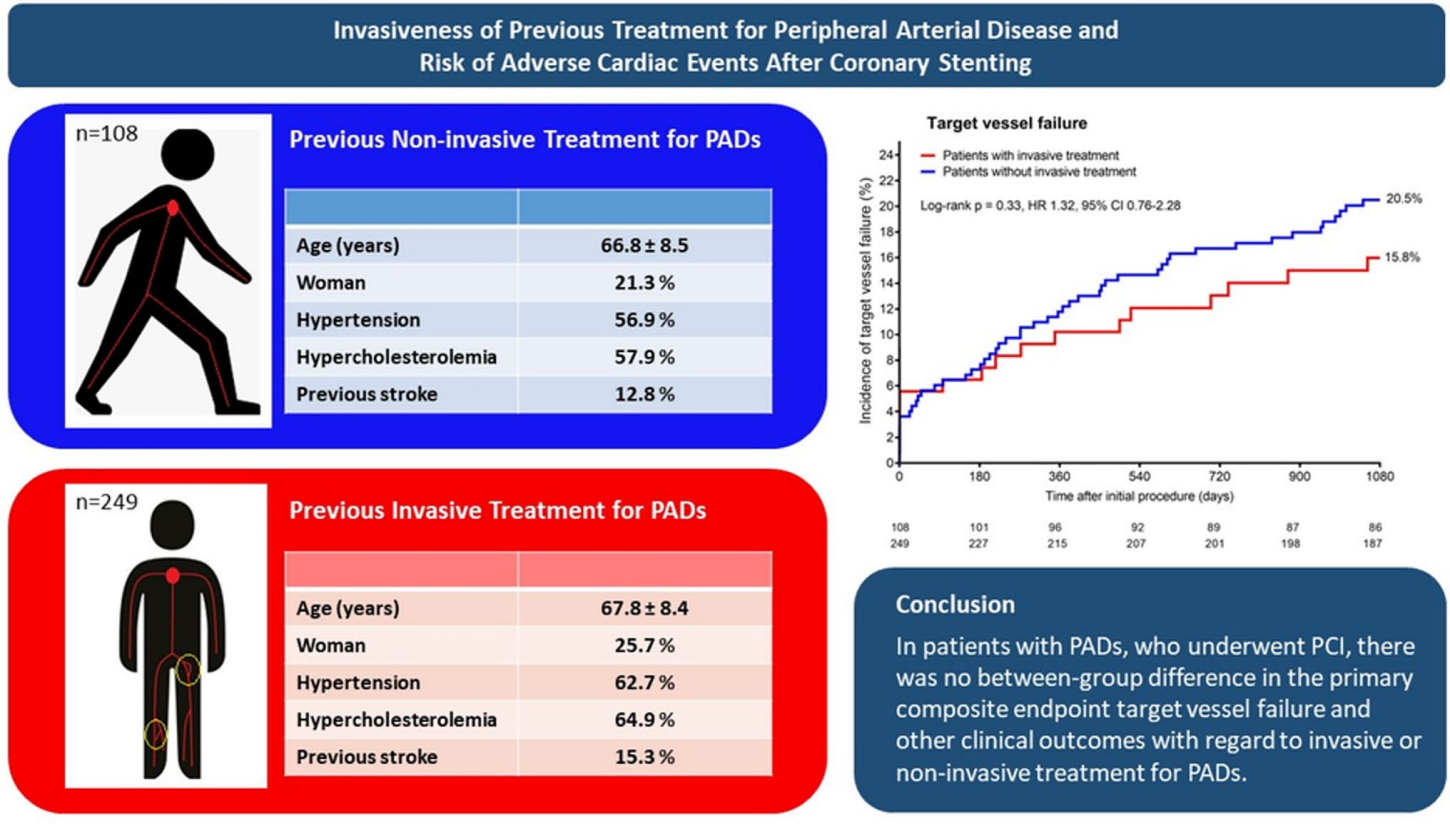

Comparison of patients with non-invasive and invasive PADs treatment. *PADs* peripheral arterial disease, *PCI* percutaneous coronary intervention.

**Supplementary Information:**

The online version contains supplementary material available at 10.1007/s12928-024-00986-7.

## Introduction

Atherosclerosis is a disease that can lead to progressive luminal obstruction, vascular occlusion, or aneurysmal dilation in affected arteries. In patients with polyvascular disease, atherosclerosis simultaneously affects more than one vascular region, such as the coronary arteries, the arteries of the lower limbs, carotid or mesenteric arteries, and the aorta [1]. In patients with coronary artery disease, the prevalence of concomitant peripheral arterial disease (PADs) has been associated with a larger coronary artery disease burden [2], worse cardiac function [3], and an unfavorable prognosis [4, 5]. Of all patients undergoing percutaneous coronary intervention (PCI), 5–10% have concomitant PADs [6–11], which has been shown to increase the risk of mortality, ischemic coronary events, and repeated coronary revascularization [11–14]. This association between concomitant PADs and increased event risk after PCI was not only seen after coronary treatment with bare metal or early-generation drug-eluting stents but also after treating all-comer patients with contemporary drug-eluting stents [15].

Information on the invasiveness of treatment for PADs might help to identify PCI patients at particularly high risk for adverse events, which could be useful during Heart Team discussions or when informing patients about their procedure-related risk. Yet, no data have been published about the impact of previous invasive versus non-invasive treatment of PADs on long-term outcomes after PCI. Therefore, we pooled patient-level data from four randomized PCI all-comer trials and classified patients with concomitant PADs based on the previous invasiveness of PADs treatment (i.e., invasive or non-invasive). Aim of this study was to evaluate the impact of the invasiveness of previous PADs treatment on 3-year clinical outcomes following PCI. In addition, we assessed the impact of clinical characteristics, such as the Fontaine stage prior to PCI, on clinical outcomes after PCI.

## Methods

### Study design

Data were pooled from PCI patients with a history of concomitant PADs, enrolled in one of the TWENTE trials ((TWENTE I, *clinicaltrials.gov: NCT01066650*), DUTCH PEERS (TWENTE II, *NCT01331707*), BIO-RESORT (TWENTE III, *NCT01674803*), and BIONYX (TWENTE IV, *NCT02508714*)). The trials enrolled all-comer patients who required PCI with drug-eluting stents for the treatment of any chronic or acute coronary syndromes (TWENTE II-IV), except for the first trial (TWENTE I) which did not include patients with ST-segment elevation myocardial infarction during the last 48 h before the index PCI. Protocols of all four studies have been published previously [16–19]. The inclusion criteria were broad and patients were eligible for participation if they were at least 18 years old and capable of providing informed consent, and if they had an estimated life expectancy of 12 months and no planned surgery during the next few months. The four trials were approved by the Medical Ethics Committee Twente and the Institutional Review Boards of all participating centers. In addition, the trials complied with the Declaration of Helsinki, and all study participants provided written informed consent.

Of all PADs patients who underwent PCI at our tertiary specialized center (Medisch Spectrum Twente, Enschede, the Netherlands), medical records were reviewed for details of their PADs treatment. Some patients, who were treated with PCI at Medisch Spectrum Twente, were treated for PADs by the vascular surgeon in another hospital or by the general practitioner. Patients who had been enrolled at other PCI centers were not considered for this analysis, as information on the treatment for PADs was generally not available.

Patients with concomitant PADs were classified, based on how PADs had been treated: invasive versus non-invasive. All previous treatments for PADs were assessed for this purpose. In a part of the patients, detailed information on the specific PADs treatment was available from a clinical PADs registry, managed by vascular specialists of our hospital.

### Procedures, follow-up, and clinical event adjudication

PCI procedures were performed according to standard techniques. Type and duration of antiplatelet therapy and choice of concomitant medication were based on routine clinical practice, current international guidelines, and the operator’s judgment. The technical details of the implanted new-generation drug-eluting stents have been reported previously [16–19]. In the case of suspected ischemia, electrocardiographs and cardiac biomarkers were systematically assessed with subsequent serial measurements. Via questionnaires, patient visits to outpatient clinics, or telephone-based follow-up, information on medication and adverse events were obtained. Foundation Cardiovascular Research and Education Enschede (Enschede, the Netherlands) performed trial and data management, data monitoring was performed by an independent clinical research organization, and independent clinical event committees adjudicated the adverse clinical events using the same definitions in all four trials [16–19].

### Definitions

The primary composite endpoint of the individual trials and the current analysis was target vessel failure (TVF), a composite of cardiac mortality, target vessel-related myocardial infarction, or clinically indicated target vessel revascularization. Secondary endpoints included the following two composite endpoints and their individual components: major adverse cardiac events (MACE; all-cause mortality, any myocardial infarction, emergent coronary bypass surgery, or clinically indicated target lesion revascularization), and target lesion failure (cardiac mortality, target vessel myocardial infarction, or clinically indicated target lesion revascularization). Clinical endpoints were defined according to the Academic Research Consortium [20, 21].

Trial participants were classified as having peripheral arterial disease if they –by anamnesis or medical record– had a history of: symptomatic atherosclerotic lesion in the lower or upper extremities; atherosclerotic lesion in the aorta causing symptoms or requiring treatment; atherosclerotic lesion in the carotid or vertebral arteries related to a non-embolic ischemic cerebrovascular event; or symptomatic atherosclerotic lesion in a mesenteric artery [22, 23].

Participation in an exercise program or treatment with medication only was classified as a *non-invasive* therapeutic approach for PADs. Amputation, endarterectomy, bypass surgery, percutaneous transluminal angioplasty, and stenting were classified as *invasive* treatment modalities. Patients who received invasive as well as non-invasive treatment were classified as patients with invasive treatment.

### Statistical analysis

We compared demographics, angiographic characteristics, and clinical outcomes of patients with invasive and non-invasive treatment for PADs. For dichotomous and categorical variables, data were expressed as frequencies with percentages. Continues variables were reported as mean ± standard deviation (SD). Differences in categorical variables were assessed by Chi-square test and differences in continuous variables were assessed with the Student’s *t*-test. Kaplan–Meier methods were used to assess time to the endpoints and the p-value of the log-rank test was used for between-group comparisons. Cox proportional hazards analysis was used to compute hazard ratios with 2-sided confidence intervals. Statistical analyses were performed with SPSS software (version 28, IBM, Armonk, NY). *P*-values and confidence intervals were two-sided, and *p*-values < 0.05 were considered significant.

## Results

**Fig. 1:**
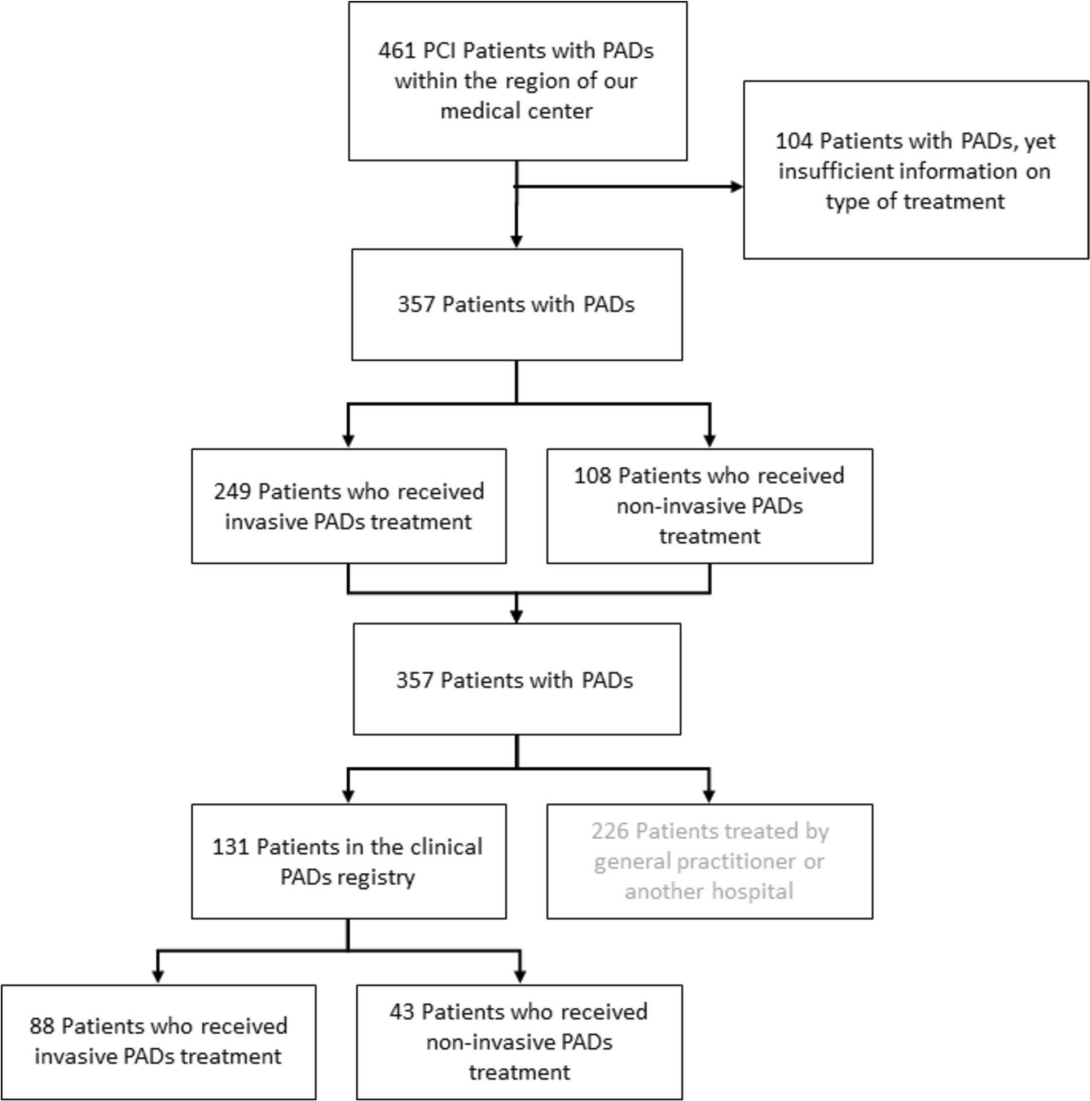
Study flowchart. The number of patients with symptomatic PADs and information about the type of treatment of PADs. *PADs* peripheral arterial disease

Of all 461 PCI patients with concomitant PADs, referred from our region, detailed information on previous PADs treatment was available in 357 (77.4%) patients, who represented the study population (Fig. 1). Of these patients, 249 (69.7%) had received invasive treatment for PADs and 108 (30.3%) had non-invasive treatment. Both patient groups did not significantly differ in baseline demographics and clinical characteristics (Table 1). Furthermore, there was neither a difference in clinical syndromes at the time of the index PCI procedure nor in procedural characteristics (Table 1).

**Table 1:** Baseline demographical, clinical and procedural characteristics of patients with and without invasive treatment for peripheral arterial disease

Characteristics	Invasive treatment		*p* value
	Yes (*n* = 249)	No (*n* = 108)	
**Baseline characteristics**			
Age (years)	67.8 ± 8.4	66.8 ± 8.5	0.30
Woman	64 (25.7)	23 (21.3)	0.37
Body-Mass Index (kg/m^2^)	27.3 ± 4.0	27.6 ± 4.6	0.51
Smoker	75/240 (31.3)	39/107 (36.4)	0.34
Diabetes mellitus	74 (29.7)	32 (29.6)	0.99
Renal failure*	25 (10.0)	10 (9.3)	0.82
Hypertension	156 (62.7)	62 (57.4)	0.35
Hypercholesterolemia	159 (64.9)	61 (57.5)	0.19
Previous stroke	38 (15.3)	14 (13.0)	0.57
LVEF < 30%	16/240 (6.7)	4/106 (3.8)	0.29
Family history of coronary artery disease	135/236 (57.2)	59/104 (56.7)	0.94
Previous myocardial infarction	66 (26.5)	34 (31.5)	0.34
Previous percutaneous coronary intervention	69 (27.7)	27 (25.0)	0.60
Previous coronary bypass surgery	42 (16.9)	18 (16.7)	0.96
Clinical syndrome at presentation			0.45
Stable angina pectoris	111 (44.6)	49 (45.4)	
STEMI	22 (8.8)	9 (8.3)	
Non-STEMI	56 (22.5)	31 (28.7)	
Unstable angina pectoris	60 (24.1)	19 (17.6)	
**Percutaneous coronary intervention: Procedural characteristics**			
Multivessel treatment	56 (22.5)	30 (27.8)	0.28
Target vessels			
Left main stem	13 (5.2)	3 (2.8)	0.31
Right coronary artery	118 (47.4)	49 (44.4)	0.61
Left anterior descending artery	85 (34.1)	44 (40.7)	0.23
Left circumflex artery	82 (32.9)	36 (33.3)	0.94
Bypass graft	14 (5.6)	9 (8.3)	0.34
Length of stent (mm)	47.3 ± 34.4	47.6 ± 28.4	0.91
Calcified lesion treated	76 (30.5)	35 (32.4)	0.72
Ostial lesion treatment	34 (13.7)	12 (11.1)	0.51
Bifurcation treatment †	73 (29.3)	27 (25.0)	0.40
Chronic total occlusion treatment	15 (6.0)	9 (8.3)	0.42
**Medication after PCI**			
Acetylsalicylic acid	242 (97.2)	107 (99.1)	0.27
Ticagrelor	40 (16.1)	18 (16.7)	0.89
Prasugrel	3 (1.2)	1 (0.9)	0.82
Clopidogrel	204 (81.9)	89 (82.4)	0.91

Values are mean ± SD, *n* (%) or *n*/*N* (%). Procedures present patient-level data

*Defined as previous renal failure, creatinine ≥ 130 μmol/L, or the need for dialysis

^†^Target lesions were classified as bifurcated if a side branch ≥ 1.5 mm originated from them

*LVEF* Left ventricle ejection fraction, *non-STEMI* non–ST-segment–elevation myocardial infarction, *STEMI* ST-segment–elevation myocardial infarction

**Fig. 2:**
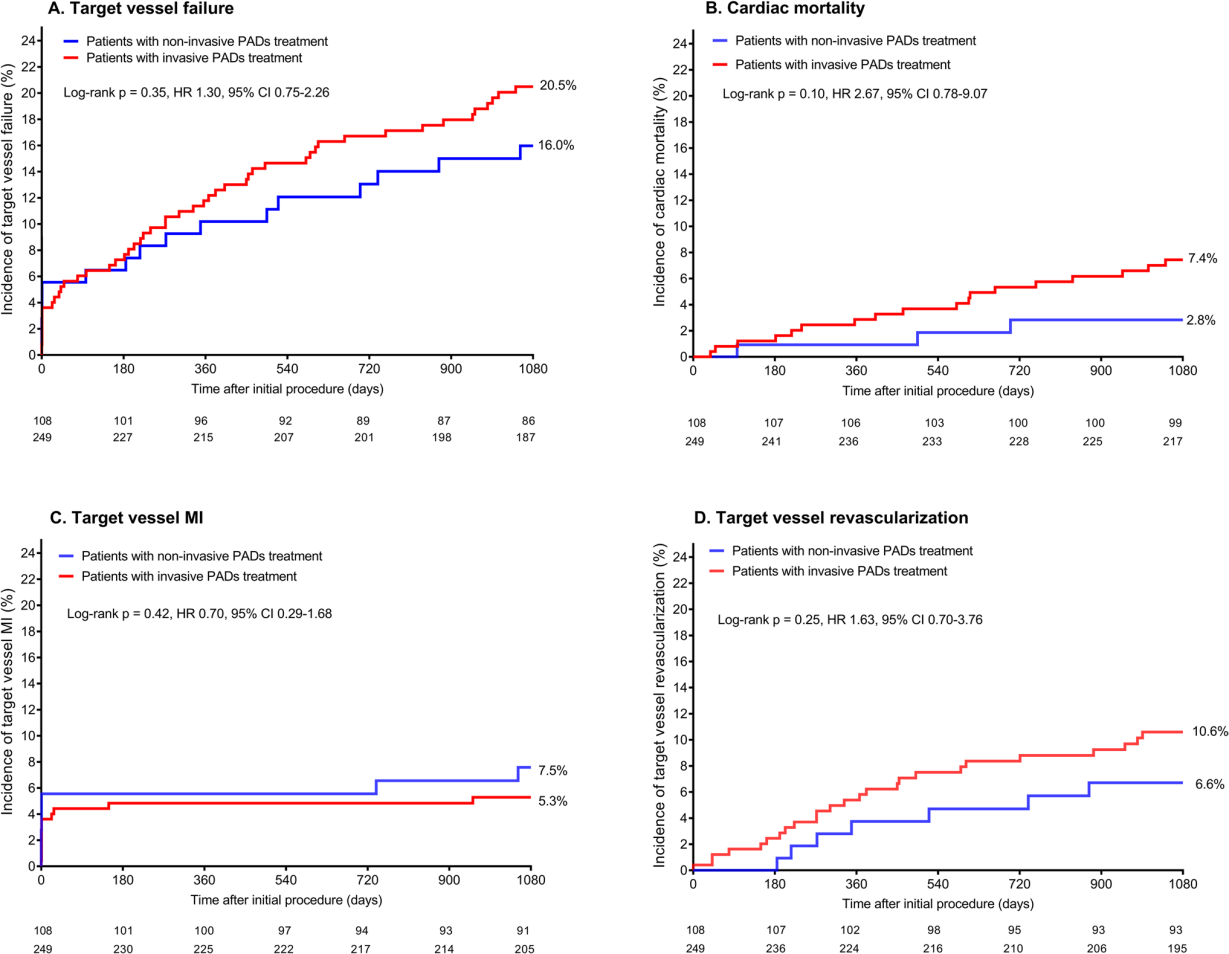
Kaplan–Meier cumulative event curves for the endpoint target vessel failure and its individual components at 3-year follow-up. Kaplan–Meier cumulative incidence curves for: (**A**) the endpoint target vessel failure, a composite of cardiac mortality (**B**), target vessel-related myocardial infarction (**C**), or clinically driven target vessel revascularization (**D**). Patients with (red) and without (blue) invasive treatment for peripheral arterial disease. *HR* hazard ratio, *MI* myocardial infarction, *PADs* peripheral arterial disease

The clinical outcome after PCI of patients with invasive and non-invasive treatment for PADs is presented in Table 2. At 3-year follow-up, the primary endpoint of TVF was met by 50 of the 249 (20.5%) patients with invasive treatment for PADs and by 17 of the 108 (16.0%) patients with non-invasive treatment (HR: 1.30, 95% CI 0.75–2.26, *p* = 0.35; Fig. 2). Furthermore, although many hazard ratios appeared to be higher, there was no statistically significant difference in the secondary endpoints: all-cause mortality (HR: 1.48, 95% CI 0.70–3.13, *p* = 0.30); cardiac mortality (HR: 2.67, 95% CI 0.78–9.07, *p* = 0.10); target vessel revascularization (HR: 1.63, 95% CI 0.70–3.76, *p* = 0.25); target lesion failure (HR: 1.29, 95% CI 0.72–2.32, *p* = 0.39); and MACE (HR: 1.16, 95% CI 0.71–1.90, *p* = 0.55; Table 2). In patients with invasive treatment for PADs, the rate of any myocardial infarction was numerically lower than in patients with non-invasive treatment, but this difference was statistically not significant (HR: 0.69, 95% CI 0.31–1.52, *p* = 0.35).

**Table 2:** Clinical outcomes at 3-year in patients with and without invasive treatment for peripheral arterial disease

Outcome	Invasive treatment		HR (95%-CI)	P_log-rank_
	Yes (*n* = 249)	No (*n* = 108)		
Target vessel failure*	50 (20.5)	17 (16.0)	1.30 (0.75–2.26)	0.35
All-cause mortality	30 (12.1)	9 (8.3)	1.48 (0.70–3.13)	0.30
Cardiac mortality	18 (7.4)	3 (2.8)	2.67 (0.78–9.07)	0.10
Any myocardial infarction	16 (6.6)	10 (9.6)	0.69 (0.31–1.52)	0.35
Target vessel related myocardial infarction	13 (5.3)	8 (7.6)	0.70 (0.29–1.68)	0.42
Target lesion failure†	44 (18.0)	15 (14.1)	1.29 (0.72–2.32)	0.39
Target vessel revascularization	25 (10.6)	7 (6.6)	1.63 (0.70–3.76)	0.25
Target lesion revascularization	17 (7.2)	4 (3.7)	1.92 (0.65–5.71)	0.23
Definite stent thrombosis	1 (0.4)	0		
Major adverse cardiac events‡	58 (23.3)	22 (20.4)	1.16 (0.71–1.90)	0.55

Data are *n* (%)

*The endpoint of target vessel failure is a composite of cardiac mortality, target vessel-related myocardial infarction, and clinically indicated target vessel revascularization

^†^Target lesion failure is a composite of cardiac mortality, target vessel-related myocardial infarction, and clinically indicated target lesion revascularization

^‡^Major adverse cardiac events is a composite of all-cause mortality, any myocardial infarction, emergent coronary artery bypass surgery, and clinically indicated target lesion revascularization

*HR* hazard ratio, *CI* confidence interval

Highly detailed information on the specific PADs treatment was available for 131 study patients who also participated in a dedicated clinical PADs registry, managed by the vascular specialists of our center; 88 (67.2%) of these patients were invasively treated and 43 (32.8%) non-invasively. Baseline demographics and PCI procedural characteristics did not differ between patient groups, except for Fontaine stages and were similar to all study patients (Supplemental Table 1). In accordance with the findings in the total study population, the two treatment-based patient groups of the clinical PADs registry showed at 3-year follow-up no statistically significant difference in TVF (HR: 1.31, 95%-CI: 0.47–3.67, *p* = 0.61) or any secondary endpoint (Table 3). Of the non-invasive treatment group, 41 (95.3%) patients followed a supervised exercise program. Of the invasive treatment group, 31 (35.2%) patients underwent percutaneous transluminal angioplasty, 12 (13.6%) had endarterectomy, 9 (10.2%) were treated with bypass surgery, and 30 (34.1%) underwent surgery plus percutaneous transluminal angioplasty (Supplemental Table 2).

**Table 3:** Clinical outcomes at 3-year in patients with and without invasive treatment for peripheral arterial disease included in the clinical PADs registry

Outcome	Invasive treatment		HR (95%-CI)	P_log-rank_
	Yes (*n* = 88)	No (*n* = 43)		
Target vessel failure*	13 (15.1)	5 (11.6)	1.31 (0.47–3.67)	0.61
All-cause mortality	9 (10.2)	3 (7.0)	1.51 (0.41–5.59)	0.53
Cardiac mortality	3 (3.6)	1 (2.3)	1.53 (0.16–14.72)	0.71
Any myocardial infarction	6 (6.9)	2 (4.7)	1.45 (0.29–7.16)	0.65
Target vessel related myocardial infarction	6 (6.9)	2 (4.7)	1.45 (0.29–7.16)	0.65
Target lesion failure†	12 (13.9)	4 (9.3)	1.51 (0.49–4.67)	0.47
Target vessel revascularization	7 (8.2)	2 (4.7)	1.79 (0.37–8.60)	0.46
Target lesion revascularization	5 (5.9)	1 (2.3)	2.54 (0.30–21.75)	0.38
Definite stent thrombosis	1 (1.2)	0		
Major adverse cardiac events‡	18 (20.5)	6 (14.0)	1.52 (0.60–3.82)	0.37
Bleeding	4/46 (8.8)	3/28 (10.7)	0.82 (0.18–3.64)	0.79

Data are *n* (%)

*The endpoint of target vessel failure is a composite of cardiac mortality, target vessel-related myocardial infarction, and clinically indicated target vessel revascularization

^†^Target lesion failure is a composite of cardiac mortality, target vessel-related myocardial infarction, and clinically indicated target lesion revascularization

^‡^Major adverse cardiac events is a composite of all-cause mortality, any myocardial infarction, emergent coronary artery bypass surgery, and clinically indicated target lesion revascularization

*HR* hazard ratio, *CI* confidence interval

Among the patients who were also included in the clinical PADs registry, Fontaine stages were higher in patients who received invasive treatment for PADs than in patients who received non-invasive treatment (*p* < 0.001; Supplemental Table 1). In addition, Supplemental Table 2 presents the frequencies by which different PADs treatment modalities were applied stratified by the Fontaine stage. Patients with a pain-free walking distance > 200 m (Fontaine stage IIa) had a lower all-cause mortality than patients with rest pain (Fontaine stage III; Supplemental Table 3). Invasive treatment of carotid artery obstructions was performed in 13 patients who were found to have a higher risk of myocardial infarction, target lesion revascularization, and target vessel and target lesion failure than 118 patients who were known to have been treated for non-carotid PADs (Supplemental Table 4).

## Discussion

### Main findings

Of the total study population of 357 PCI patients with concomitant PADs, 70% had invasive treatment for PADs while 30% was treated non-invasively. These two patient groups, which turned out not to differ in demographics, and clinical and PCI procedural characteristics, showed at 3-year follow-up no statistically significant difference in the primary clinical endpoint TVF (20.5% vs. 16.0%), the secondary endpoint MACE (23.3% vs. 20.4%), and all other secondary endpoints. All-cause mortality (12.1% vs. 8.3%) and cardiac mortality (7.4% vs. 2.8%) rates were numerically higher in study patients with previous invasive PADs treatment; yet, these dissimilarities did not reach statistical significance, possibly due to sample size. Highly detailed information on previous PADs treatment was available in 131 patients who also participated in a dedicated clinical PADs registry. Baseline characteristics and long-term outcome following PCI of patients who previously had invasive treatment for PADs were found to be similar in patients with an exclusively non*-*invasive treatment for PADs. Furthermore, the all-cause mortality rate was higher in patients with rest pain (Fontaine stage III) than in patients with a pain-free walking distance of more than 200 m (Fontaine stage IIa).

### Previous studies

To the best of our knowledge, the present analysis is the first to assess the impact of the invasiveness of previous PADs treatment on long-term outcome after PCI. In former studies, various approaches of detecting and defining PADs have been applied [6–9, 13, 24, 25]. As a result, the distribution of treatment types for PADs differs between studies.

For instance, peripheral artery disease can be assessed by measuring the ankle-brachial index, but usually only 10–30% of all patients with a decreased ankle-brachial index report symptoms consistent with classic claudication [26]. When using the ankle-brachial index, it is fair to assume that 7–13% of the patients undergoing (coronary angiography or) PCI have undiagnosed PADs [24, 25]. Yet, if the presence of PADs is defined based on the assessment of medical records, only patients who currently are (or previously were) symptomatic are classified as PADs patients, while asymptomatic patients with abnormal ankle-brachial index are not. In clinical practice, when patients present with anginal symptoms, information about previous PADs treatment is generally available from the medical record, while measurements of the ankle-brachial index often are not.

Furthermore, the vascular regions included in the definition of PADs differ between studies. Many studies included not only atherosclerotic disease in the lower limb but also cerebrovascular disease [8, 10], aortic pathologies [6, 7, 27], or atherosclerotic disease in all non-coronary arteries except for the aorta [9, 13]. Such between-study differences in the definition of PADs result in substantial differences between the respective study populations, which may lead to dissimilar findings when assessing the potential impact of PADs on clinical outcomes after PCI.

Previously, PADs with a decreased ankle-brachial index were associated with an increased risk of cardiovascular and all-cause mortality, ischemic or hemorrhagic stroke, repeated target lesion revascularization, major bleeding, and major adverse cardiac and cerebrovascular events at follow-up with a duration of 1 to 4 years [24, 28, 29]. Even in patients with borderline decreased ankle-brachial index, higher event rates were observed 18 months after PCI for the composite endpoint of major adverse cardiac and cerebrovascular events as well as for stroke [30].

Studies that defined PADs based on data from the medical history (rather than ankle-brachial index-measurements) showed quite similar results [9, 11, 15, 24, 28, 29]. A previous analysis of PADs patients from a series of four randomized PCI trials found that patients with a history of PADs, based on medical records, had higher 3-year risks of target vessel failure, repeated target vessel revascularization, MACE, and all-cause mortality than patients without PADs [15]. The findings of that analysis are in accordance with former studies that evaluated clinical outcomes after PCI in patients with PADs based on medical records. In those studies, the presence of PADs was associated with lower rates of procedural success [1, 9] and with higher in-hospital and 2-year rates of stroke, myocardial infarction, MACE, clinically relevant bleeding, and mortality [1, 6, 9–12, 27]. In addition, PCI patients with concomitant PADs had higher all-cause mortality rates at long-term follow-up after PCI [6, 10].

Hence, regardless of the way of defining PADs, PCI patients with PADs had a worse clinical outcome than those without PADs. Although the aforementioned studies included somewhat different patient populations with varying treatments for PADs, the presence of PADs had a similar, unfavorable impact on long-term clinical outcome following PCI. In the present study in PCI patients with concomitant PADs, patients with a history of invasive treatment had on average a higher (maximum) Fontaine stage than patients with exclusively non-invasive treatment for PADs, as may be expected. Yet, between PCI patients with invasive and non-invasive previous treatment for concomitant PADs, we found no significant difference in clinical outcome after PCI.

### Implications

While PADs itself may directly account for some post-PCI cardiovascular events and mortality, the presence of PADs can be seen as an indication of greater plaque burden and more progressive atherosclerotic disease in the vasculature, including the coronary arteries [1, 2, 14]. The findings of the present study suggest that the presence of PADs –regardless of the invasiveness of previous PADs treatment– should be seen as a marker of an increased event risk after a PCI. Moreover, among PCI patients with concomitant PADs, patients with a higher Fontaine stage may show a higher mortality than PADs patients with a pain-free walking distance of more than 200 m (Fontaine stage IIa). While detailed information on PADs location and previous treatment is valuable when choosing a vascular access site for PCI, the present study shows that high-risk patients for PCI can be identified by straightforwardly consulting the medical records, searching for known PADs irrespective of the invasiveness of PADs treatment. Knowledge of these findings may be particularly useful when considering PCI during Heart Team discussions, and when informing patients about their individual adverse event risk.

### Limitations

The results of the present post-hoc study should be considered hypothesis-generating. Nevertheless, this study is the first to assess the potential impact of previous PADs treatment on clinical outcome after PCI with contemporary drug-eluting stents. Pooled individual patient-level data from four PCI all-comer trials were studied, as adverse event rates were relatively low. The relatively low adverse event rates may reflect progress in coronary stent design and concomitant pharmacological therapy (including antithrombotic strategy), rather than missing adverse events in these randomized clinical trials with high follow-up, external monitoring, and independent event adjudication. Of all PADs patients who underwent PCI at the Medisch Spectrum Twente (Enschede, the Netherlands), medical records were reviewed for details of their PADs treatment. Of patients included in the other hospitals detailed information about PADs was not available. In addition, information about PADs type, symptoms, and treatment was unavailable in some trial participants. In addition, the patient group with non-invasive treatment for PADs may be somewhat heterogeneous, as not only patients with mild PADs may have been included in this group but also some patients who were too frail to undergo invasive treatment. The collection of detailed data on anatomical PADs severity could have been of interest, but in many study patients such details were not available.

Furthermore, as a result of the PADs definition used, undiagnosed PADs have been missed. Assessing the ankle-brachial index in all PCI patients could have provided further insights. Yet, one should not expect from a randomized PCI trial that routine measurements of the ankle-brachial index are performed, especially not in the large-sized group of patients who underwent PCI for acute myocardial infarction. Moreover, such approach would not have reflected current routine clinical practice that all-comer PCI trials typically strive to emulate.

## Conclusions

In PADs patients participating in PCI all-comer trials, we found no significant relation between the invasiveness of previous PADs treatment and 3-year outcome after PCI. Consequently, high-risk patients for PCI can be identified by consulting medical records, searching for known PADs, irrespective of the invasiveness of previous PADs treatment.

### Supplementary Information

Below is the link to the electronic supplementary material.

Supplementary file1 (PDF 188 KB)
