# Supplementary material for: Invasiveness of previous treatment for peripheral arterial disease and risk of adverse cardiac events after coronary stenting
Source: Cardiovasc Interv Ther. 2024 Feb 14;39(2):173–82. doi: 10.1007/s12928-024-00986-7 (PMC10940370; doi:10.1007/s12928-024-00986-7)
Supplement: Supplementary file 1 — Supplementary file1 (PDF 188 KB) [file 12928_2024_986_MOESM1_ESM.pdf]

Invasiveness of Previous Treatment for Peripheral Arterial Disease and Risk of Adverse Cardiac Events After Coronary Stenting

Tineke H. Pinxterhuis MD[a,b], Clemens von Birgelen MD PhD[a,b], Robert H. Geelkerken MD PhD[c,d], Carine J.M. Doggen PhD[b], Theo P. Menting, MD PhD[c], K. Gert van Houwelingen, MD[a], Gerard C.M. Linssen MD PhD[e], Eline H. Ploumen MD PhD[a,b]

a. Department of Cardiology, Thoraxcentrum Twente, Medisch Spectrum Twente, Enschede, the Netherlands
b. Department of Health Technology and Services Research, Faculty BMS, Technical Medical Centre, University of Twente, Enschede, the Netherlands
c. Department of Vascular Surgery, Medisch Spectrum Twente, Enschede, the Netherlands
d. Department of Multi-modality Medical Imaging (M3I) group, Faculty of Science and Technology, Technical Medical Centre, University of Twente, Enschede, the Netherlands
e. Department of Cardiology, Ziekenhuisgroep Twente, Almelo and Hengelo, the Netherlands

SUPPLEMENTARY MATERIAL

Index

Table A1: Baseline and procedural characteristics of patients with and without invasive treatment for peripheral arterial disease included in the MST vascular registry ___________ page 2

Table A2: Fontaine classification and treatment of patients ___________________________ page 5

Table A3: Three-year clinical outcomes of peripheral arterial disease patients based on Fontaine classification ____ page 6

Table A4: Three-year clinical outcomes of peripheral arterial disease patients with and without treatment of the carotid artery included in the MST vascular registry_____________ page 8

Table A1: Baseline demographical, clinical and procedural characteristics of patients with and without invasive treatment for peripheral arterial disease included in the clinical PADs registry

	Clinical PADs registry			All PADs patients		
	Invasive treatment		p-value	Invasive treatment		p-value
	Yes (n=88)	No (n=43)		Yes (n=249)	No (n=108)	
Baseline characteristics						
Age (years)	68.0 ± 7.8	67.1 ± 7.1	0.53	67.8 ± 8.4	66.8 ± 8.5	0.30
Woman	22 (25.0)	7 (16.3)	0.26	64 (25.7)	23 (21.3)	0.37
Body-Mass Index (kg/m^2)	27.4 ± 3.8	27.7 ± 3.6	0.67	27.3 ± 4.0	27.6 ± 4.6	0.51
Smoker	32/86 (37.2)	15/42 (35.7)	0.87	75/240 (31.3)	39/107 (36.4)	0.34
Diabetes mellitus	25 (28.4)	11 (25.6)	0.73	74 (29.7)	32 (29.6)	0.99
Renal failure *	9 (10.2)	2 (4.7)	0.28	25 (10.0)	10 (9.3)	0.82
Hypertension	55 (62.5)	26 (60.5)	0.82	156 (62.7)	62 (57.4)	0.35
Hypercholesterolemia	59 (67.0)	24 (55.8)	0.21	159 (64.9)	61 (57.5)	0.19
Previous stroke	14 (15.9)	6 (14.0)	0.77	38 (15.3)	14 (13.0)	0.57
LVEF <30%	6 (7.1)	1 (2.3)	0.27	16/240 (6.7)	4/106 (3.8)	0.29
Family history of coronary artery disease	50 (58.8)	21 (52.5)	0.51	135/236 (57.2)	59/104 (56.7)	0.94
Previous myocardial infarction	18 (20.5)	12 (27.9)	0.34	66 (26.5)	34 (31.5)	0.34
Previous percutaneous coronary intervention	24 (27.3)	12 (27.9)	0.94	69 (27.7)	27 (25.0)	0.60
Previous coronary bypass surgery	16 (18.2)	6 (14.0)	0.54	42 (16.9)	18 (16.7)	0.96
Clinical syndrome at presentation			0.65			0.45

Stable angina pectoris	42 (47.7)	18 (41.9)		111 (44.6)	49 (45.4)	
STEMI	5 (5.7)	5 (11.6)		22 (8.8)	9 (8.3)	
Non-STEMI	19 (21.6)	10 (23.3)		56 (22.5)	31 (28.7)	
Unstable angina pectoris	22 (25.0)	10 (23.3)		60 (24.1)	19 (17.6)	
Peripheral artery disease						
Fontaine classification			<0.001			
I (asymptomatic)	0	4/40 (10.0)				
IIa (intermittent claudication, walking distance >200)	27/80 (34.2)	29/40 (72.5)				
IIb (intermittent claudication, walking distance <200)	39/80 (49.4)	6/40 (15.0)				
III (Rest pain)	10/80 (12.5)	1/40 (2.5)				
IV (Ischaemic ulcers)	2/80 (2.5)	0				
Percutaneous coronary intervention: Procedural characteristics						
Multivessel treatment	21 (23.9)	8 (18.6)	0.50	56 (22.5)	30 (27.8)	0.28
Target vessels						
Left main stem	8 (9.1)	1 (2.3)	0.15	13 (5.2)	3 (2.8)	0.31
Right coronary artery	36 (40.9)	19 (44.2)	0.72	118 (47.4)	49 (44.4)	0.61
Left anterior descending artery	32 (36.4)	15 (34.9)	0.87	85 (34.1)	44 (40.7)	0.23
Left circumflex artery	30 (34.1)	15 (34.9)	0.93	82 (32.9)	36 (33.3)	0.94
Bypass graft	5 (5.7)	2 (4.7)	0.81	14 (5.6)	9 (8.3)	0.34

Length of stent (mm)	50.9 ± 36.0	44.9 ± 31.6	0.35	47.3 ± 34.4	47.6 ± 28.4	0.91
Calcified lesion treated	33 (37.5)	15 (34.9)	0.77	76 (30.5)	35 (32.4)	0.72
Ostial lesion treatment	12 (13.6)	5 (11.6)	0.75	34 (13.7)	12 (11.1)	0.51
Bifurcation treatment †	28 (31.8)	9 (20.9)	0.19	73 (29.3)	27 (25.0)	0.40
Chronic total occlusion treatment	5 (5.7)	4 (9.3)	0.44	15 (6.0)	9 (8.3)	0.42

Values are mean ± SD, n (%) or n/N (%). Procedures present patient-level data. *Defined as previous renal failure, creatinine ≥130 µmol/L, or the need for dialysis; †Target lesions were classified as bifurcated if a side branch ≥1.5 mm originated from them.

Abbreviations: LVEF=Left ventricle ejection fraction; non-STEMI=non-ST-segment-elevation myocardial infarction; PADs= peripheral arterial disease; STEMI=ST-segment-elevation myocardial infarction.

Table A2: Fontaine classification and treatment of patients

Fontaine	Asymptomatic (n=6)	Walking distance >200 (n=56)	Walking distance <200 (n=45)	Rest pain (n=11)	Ischemic ulcers (n=2)	No class (n=11)
**Non-invasive treatment**						
Supervised exercise program	4	28	6	1	0	2
Medical treatment only	1	0	0	0	0	1
**Invasive treatment**						
PTA	1	11	14	4	0	1
Surgery						
Bypass	0	2	3	2	0	2
Endarterectomy	0	5	5	0	0	2
Bypass + endarterectomy	0	3	1	0	1	1
Surgery + PTA	1	8	14	4	1	2

Data are n (%). *Abbreviations:* PTA= percutaneous transluminal angioplasty

Table A3: Three-year clinical outcomes of peripheral arterial disease patients based on Fontaine classification

Outcome	Fontaine classification					HR (95%-CI) IIa vs. IIb	$P_{log-rank}$ IIa vs. IIb	HR (95%-CI) IIb vs. III	$P_{log-rank}$ IIb vs. III	HR (95%-CI) IIa vs. III	$P_{log-rank}$ IIa vs. III
	Fontaine I (n=4)	Fontaine IIa (n=56)	Fontaine IIb (n=45)	Fontaine III (n=12)	Fontaine IV (n=2)						
Target vessel failure *	1 (25)	7 (12.5)	8 (18.4)	1 (8.3)	0	1.46 (0.53-4.03)	0.46	0.49 (0.06-3.58)	0.44	0.64 (0.08-5.24)	0.68
All-cause mortality	1 (25)	3 (5.4)	5 (11.1)	3 (25)	0	2.19 (0.52-9.18)	0.27	2.29 (0.55-9.60)	0.24	5.25 (1.06-26.03)	*0.023*
Cardiac mortality	0	1 (1.8)	2 (4.8)	1 (8.3)	0	2.66 (0.24-29.29)	0.41	1.96 (0.18-21.64)	0.58	5.11 (0.32-81.97)	0.20
Any myocardial infarction	0	4 (7.1)	3 (6.8)	0	0	0.92 (0.21-4.11)	0.91				
Target vessel related myocardial infarction	0	4 (7.1)	3 (6.8)	0	0	0.92 (0.21-4.11)	0.91				
Target lesion failure †	0	7 (12.5)	7 (16.1)	1 (8.3)	0	1.27 (0.45-3.62)	0.65	0.52 (0.06-4.21)	0.53	0.64 (0.08-5.24)	0.68

Target vessel revascularization	1 (25)	2 (3.6)	6 (13.9)	0	0	4.05 (0.82-20.08)	0.06				
Target lesion revascularization	0	2 (3.6)	4 (9.3)	0	0	2.68 (0.49-14.61)	0.24				
Definite stent thrombosis	0	0	1 (2.3)	0	0						
Major adverse cardiac events ‡	1 (25)	9 (16.1)	10 (22.2)	3 (25)	0	1.42 (0.58-3.48)	0.44	1.07 (0.30-3.90)	0.91	1.53 (0.41-5.65)	0.52

Data are n (%). *The endpoint target vessel failure is a composite of cardiac mortality, target vessel related myocardial infarction, and clinically indicated target vessel revascularization. †Target lesion failure is a composite of cardiac mortality, target vessel related myocardial infarction, and clinically indicated target lesion revascularization. ‡Major adverse cardiac events is a composite of all-cause mortality, any myocardial infarction, emergent coronary artery bypass surgery, and clinically indicated target lesion revascularization.

Abbreviations: HR=hazard ratio; CI=confidence interval.

Table A4: Three-year clinical outcomes of peripheral arterial disease patients with or without treatment of the carotid artery included in the clinical PADs registry

Outcome	Treatment carotid artery		HR (95%-CI)	$P_{log\text{-}rank}$
	Yes (n=13)	No (n=118)		
Target vessel failure *	4 (30.8)	14 (12.1)	2.98 (0.98-9.06)	*0.042*
All-cause mortality	0	12 (10.2)		
Any myocardial infarction	5 (23.1)	3 (4.3)	5.49 (1.31-22.97)	*0.008*
Target vessel related myocardial infarction	5 (23.1)	3 (4.3)	5.49 (1.31-22.97)	*0.008*
Target lesion failure †	4 (30.8)	12 (10.4)	3.44 (1.11-10.68)	*0.022*
Target vessel revascularization	2 (15.4)	7 (6.1)	2.77 (0.58-13.36)	0.18
Target lesion revascularization	2 (15.4)	4 (3.5)	4.82 (0.88-26.32)	*0.045*
Definite stent thrombosis	1 (7.7)	0		
Major adverse cardiac events ‡	4 (30.8)	20 (16.9)	2.12 (0.72-6.21)	0.16

Data are n (%). *The endpoint target vessel failure is a composite of cardiac mortality, target vessel related myocardial infarction, and clinically indicated target vessel revascularization. †Target lesion failure is a composite of cardiac mortality, target vessel related myocardial infarction, and clinically indicated target lesion revascularization. ‡Major adverse cardiac events is a composite of all-cause mortality, any myocardial infarction, emergent coronary artery bypass surgery, and clinically indicated target lesion revascularization.

Abbreviations: HR=hazard ratio; CI=confidence interval; PADs=peripheral arterial disease
